# Dietary Diversity and the Risk of Fracture in Adults: A Prospective Study

**DOI:** 10.3390/nu12123655

**Published:** 2020-11-27

**Authors:** Jian Zhang, Dong Liang, Ai Zhao

**Affiliations:** 1 Vanke School of Public Health, Tsinghua University, Beijing 100091, China; zhangjian92@pku.edu.cn; 2 Department of Nutrition and Food Hygiene, School of Public Health, Peking University, Beijing 100191, China; 3 China National Center for Food Safety Risk Assessment, Beijing 100021, China; liangdong@cfsa.net.cn

**Keywords:** dietary diversity, fracture, adult, prospective study

## Abstract

Nutrition plays an important and modifiable role in bone health. This study aimed to investigate the effect of dietary diversity on the risk of any type of fracture in adults. Data from the China Health and Nutrition Survey collected between waves 1997 and 2015 were used. A total of 10,192 adults aged 40 years and older were included in the analysis. Both dietary diversity score (DDS) based on Chinese dietary guidelines (DDS-CDG) and minimum dietary diversity for women (DDS-MDD-W) were computed. Cox proportional hazards regression models were conducted to determine the association. Stratified analyses were conducted in women by the age of fracture using the case-control study approach. In men, higher scores in both the DDS-CDG (hazard ratio (HR) 0.70, 95% CI 0.56–0.88) and DDS-MDD-W (HR 0.67, 95% CI 0.54–0.82) were associated with decreased risk of fracture, however, the associations were not significant in women (DDS-CDG: HR 0.94, 95% CI 0.79–1.12; DDS-MDD-W: HR 0.93, 95% CI 0.79–1.09). In the stratified analyses, higher DDS-CDG (odds ratio (OR) 0.74, 95% CI 0.58–0.95) and higher DDS-MDD-W (OR 0.76, 95% CI 0.60–0.95) were associated with lower risks of fracture in women aged 40 to 60 years; in women aged over 60 years, no association was observed (DDS-CDG: OR 1.10, 95% CI 0.83–1.46; DDS-MDD-W: OR 1.00, 95% CI 0.79–1.27). In summary, higher dietary diversity was associated with decreased risk of fracture in men and middle-aged women, but not in women aged over 60 years.

## 1. Introduction

Bone fractures in elderly people are often related to increased morbidity and mortality, which is an important public health issue [1,2]. In China, osteoporotic fractures put heavy burdens on both patients and society [3,4], and the incidence of osteoporotic fracture still shows steady increase [5].

Multiple factors are known to be associated with fracture, including aging, genes, nutrition, physical activity, etc. [6]. Among these factors, nutrition plays an important and modifiable role in bone health and fracture prevention and recovery [7,8]. Although some controversies exist, it has been long recognized that calcium and vitamin D are essential in the maintenance of bone health [9,10]. In addition to this, the relationship of other nutrients and foods with fracture has been widely investigated [11,12,13]. For example, a meta-analysis showed that increased intake of vegetables was associated with a lower risk of hip fracture [11]. Considering that our diet is complex, it is also important to consider the association between fracture and diet as a whole.

The dietary diversity score (DDS) is a proxy dietary quality index, which is used to assess diet quality and nutrient adequacy of individuals [14,15]. It has been proved that an increase in individual DDS is related to increased nutrient adequacy of the diet [16]. Previous studies showed that higher DDS reduced the risk of some age-related diseases, including cognitive and memory impairment [17,18], diabetes [19], and hypertension [20]. Besides, individuals with higher DDS had lower risk of death [21,22]. In contrast, research on dietary diversity and bone fracture is absent.

In this research, we prospectively investigated the association between dietary diversity and any type of bone fracture in adults aged 40 years and older using a large-scale cohort study.

## 2. Materials and Methods

### 2.1. Study Population

**Figure 1:**
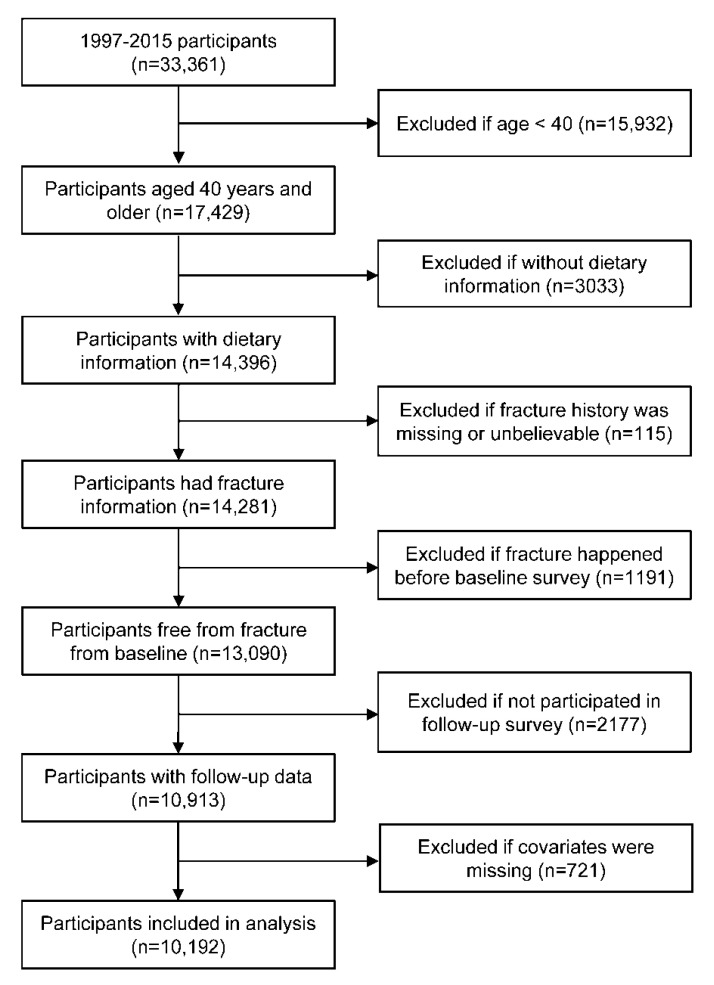
Flow chart of sample selection.

The present study was based on the China Health and Nutrition Survey (CHNS). Details of this study have been described elsewhere [23]. In brief, the CHNS is a nationwide, dynamic cohort study initiated in 1989, and follow-ups were conducted in the years 1991, 1993, 1997, 2000, 2004, 2006, 2009, 2011, and 2015. In this research, we used a sub-cohort from the CHNS. Data collected in the wave 1997 and beyond were used, and only participants aged 40 years and older were included. Participants meeting the following criteria were excluded: whose fracture information was missing or unbelievable, who only participated in the baseline survey and without follow-up data, who did not participate in dietary survey, who had fracture before the baseline survey, or who had a missing value on key covariates. The final analyses included 10,192 participants (Figure 1).

### 2.2. Ethics

The CHNS was approved by institutional review boards at the University of North Carolina at Chapel Hill and the National Institute of Nutrition and Food Safety, Chinese Center for Disease Control and Prevention. All participants gave written informed consent before they participated in the survey.

### 2.3. Assessment of Diet and Dietary Diversity Score

Both dietary recall and household food weight inventory for three consecutive days were used to record the food and beverage intake of each participant in each wave. The three survey days were randomly allocated from Monday to Sunday. Household food consumption was determined by examining changes in inventory from the beginning to the end of each day. Individual dietary intakes for the same three days were recorded by 24-h dietary recall questionnaires. Details of the dietary survey has been published elsewhere [24].

Two DDSs were used to assess the diet diversity of participants. The first DDS was developed according to Chinese dietary guidelines (CDG) [15]. The CDG included 10 food groups (cereals and tubers, vegetables, fruits, meat, soybeans and nuts, eggs, aquatic products, milk and dairy products, as well as salt and oil). Because salt and oil are necessary food groups of the Chinese diet [25], these two food groups were not included in assessing the DDS-CDG. We also referred to the minimum dietary diversity for women (DDS-MDD-W) to calculate the second DDS, which pays more attention to micronutrient intakes [26]. Although the DDS-MDD-W was developed for women aged 15 to 49 years, this score was also used in men [27]. The DDS-MDD-W included 10 food groups (starchy staples (grains, white roots and tubers, and plantains); pulses; nuts and seeds; dairy; eggs; dark green leafy vegetables; other vitamin A-rich fruits and vegetables; other vegetables; other fruits; and meat, poultry, and fish). If one participant consumed any food from a certain food group in the DDS in the past 24 h, then the participant would get one point for that food group. Total scores were 8 for DDS-CDG and 10 for DDS-MDD-W. Average daily DDSs were calculated for each participant in each wave. Cumulative average DDS across waves before the end of survey (occurrence of fracture, loss to follow-up, or the wave 2015, whichever occurred first) was used to present the long-term dietary exposure.

### 2.4. Ascertainment of Fracture

In each wave, participants were asked, “Do you have a history of bone fracture?”: no, yes, or unknown. The participants who chose “yes” were further asked, “How old were you when you had the first bone fracture?” and “How many times has this happened?” As the survey only recorded the first bone fracture age, we only included the first bone fracture in our analysis. In order to get a precise date of bone fracture, we assumed that fractures happened on 1 July of each year.

### 2.5. Assessment of Covariates

Covariates used in this study were obtained from baseline survey, including socio-demographic characteristics (age, region, residency, income, education level), lifestyles (physical activity level, alcohol consumption, and smoking status), and anthropometric measurement and disease history (body mass index (BMI) and previous diagnosis of diabetes).

Missing values for per capita household income were replaced by the median of each survey site of each wave. Income was classified into tertiles at each wave and was labeled as low, middle, or high, respectively. Participants were classified as a regular alcohol drinker if drinking alcohol every week. Physical activity assessment was in terms of metabolic equivalent of task (MET)-hours per week [28,29]. Missing values for height and weight were replaced by the mean values of two adjacent surveys. BMI was calculated as weight (kg)/(height(m))^2^.

### 2.6. Statistics

Normally distributed continuous variables were presented as means and standard deviations and were compared by *t*-test or one-way ANOVA; otherwise, medians and quartiles were used and differences between groups were compared with the Wilcoxon test. Categorical variables were presented as percentages and compared by chi-square test.

Person-years were calculated from the baseline survey date until the date when the survey ended (occurrence of fracture, loss to follow-up, or the wave 2015, whichever occurred first). Cox proportional hazards regression models were used to estimate the effect of DDS on the risk of fracture. Covariates adjusted included age at entry (continuous), region (northern or southern China), residency (urban or rural), income (low, middle, or high), education level (primary school and below, middle school, or high school and above), BMI (continuous), physical activity level (<100 or ≥100 MET-hours per week), smoking status (smoker or not), and previous diagnosis of diabetes (yes or no). Sensitivity analyses were conducted by additionally adjusting for alcohol consumption (regular drinker or not) or the wave at entry (1997, 2000, 2004, 2006, 2009, or 2011). The Cox proportional hazards regression models were conducted with the R package “survival” [30].

Since the CHNS did not record detailed information on women’s menopausal status, we did a stratified analysis by participants’ age using the case-control study approach. The case group included women who had fracture during the follow-up. All cases were divided into two groups: the first group included women whose fracture happened when they were 60 years old or younger, and the other group included those whose fracture happened after 60 years old. Controls were selected (1:4) from all cohort members free of fracture and were matched by age when fracture happened (a difference of no more than three years), region, residency, and income. The matching process was performed by a previously developed SAS macro [31]. Conditional logistic regression models were used to estimate the association between the DDS and fracture, with adjustment of education level, smoking status, physical activity level, and BMI.

All the statistics were conducted in R 4.0.2 (R Core Team (2020), Vienna, Austria) unless stated otherwise. All *p* values were two-sided, and statistical significance was defined as *p* < 0.05.

## 3. Results

### 3.1. Characteristics of Participants

Baseline characteristics of participants are shown in Table 1 and Table S1. A total of 10,192 participants were included in the study: 3877 individuals joined the cohort in the year 1997, 1137 in 2000, 1231 in 2004, 702 in 2006, 1139 in 2009, and 2106 in 2011. The average DDS-CDG of participants across waves was 3.81 ± 1.00 (ranging from 1.33 to 8.00), and the average for the DDS-MDD-W was 4.41 ± 1.09 (ranging from 1.33 to 10.00). Both in men and women, individuals living in southern China, living in urban areas, having higher education and income, who were non-smokers, having lower physical activity level, having higher BMI, and having a history of diabetes tended to have higher scores in both the DDS-CDG and DDS-MDD-W. Besides, in men, being older was associated with higher scores in both DDSs. Women aged 50 to 60 years had the highest average DDSs than the other two age groups (Table S2).

### 3.2. Association between Dietary Diversity Score and Fracture

During a median follow-up of 7.0 years (ranging from 0.5 to 18.7 years; total person-years [TPY] 91,714; TPY for men 42,330; TPY for women 49,384), 171 fractures occurred in men and 251 in women. Absolute rates were 4.04 and 5.08 fractures per 1000 person-years for men and women, respectively. Participants who reported bone fracture had relatively lower scores on animal-source food (eggs, meat, milk and dairy products, and aquatic products (only in men)), fruits and fruit subgroups, vegetable subgroups, and nuts and seeds (only in men). In women, those with fracture had higher scores on dark green leafy vegetables (Table S3).

After the adjustment of covariates, both higher DDS-CDG and higher DDS-MDD-W were associated with decreased fracture risk in men. The associations of DDS with the incidence of bone fracture were not significant in women (Table 2). In the sensitivity analyses, the associations of DDS-CDG and DDS-MDD-W with bone fracture did not change after adjustment of wave at entry or alcohol consumption (Table S4).

### 3.3. Case-Control Study in Women

Case-control studies showed that in women aged 60 years and below both the DDS-CDG and the DDS-MDD-W were inversely associated with the risk of fracture; however, in women aged over 60 years, neither score was associated with the risk of fracture (Table 3).

## 4. Discussion

To the best of our knowledge, our study is the first to investigate the effects of dietary diversity on the risk of fracture using a large-scale cohort study. Our study revealed that higher dietary diversity contributed to a lower risk of bone fracture in men and middle-aged women in China.

In our study, we found both DDS-CDG and DDS-MDD-W were associated with decreased risk of fracture in men and middle-aged women. The mechanisms linking DDS and bone fracture are unclear; however, we infer several potential pathways listed below that may contribute to this association. To begin with, compared with participants who reported fracture, those who did not had relatively higher intakes of animal-sourced food, including meat, aquatic products, eggs, and milk and dairy products. These foods provide our bodies with high quality proteins. Although the role of dietary protein in bone health is still controversial, some reviews showed that dietary protein had a beneficial effect on bone density [32,33,34]. High-protein intake may promote bone heath through its impact on calcium absorption, stimulation of the secretion of insulin-like growth factor-1, and enhancement of lean body mass [34]. Besides, milk and dairy products, which are included in both DDSs, are considered to be good for bone health [35]. Milk products are high in calcium and magnesium, which play a structural role in bone health [36], and a previous study showed milk protein could promote bone formation and suppressed bone resorption in adult men [37]. In addition, previous studies showed that oxidative stress played an important role in bone remodeling [38]. The bone continuously renews itself throughout lifecycle by the actions of osteoclasts, osteoblasts, and osteocytes, which are regulated by hormones, growth factors, and cytokines [38,39,40]. Oxidative stress activates the differentiation of preosteoclasts and induces the apoptosis of osteoblasts and osteocytes, causing an imbalance between osteoclast and osteoblast activity and loss of bone mass [38,41,42,43]. Both the DDS-CDG and the DDS-MDD-W give special attention to fruits, vegetables, legumes, and nuts, which are rich sources of antioxidants and anti-inflammatory components [44]. Our study found participants without fracture had relatively high intakes of nuts and seeds, fruits, and vegetable subgroup. Higher dietary diversity may promote bone health by countering age-related oxidative stress and inflammation [11]. Last but not least, low dietary diversity reflects a relatively poor nutritional status [21], which is also inversely associated with gastrointestinal microbiome diversity [45]. Many studies have already revealed that the gut microbiome plays a crucial role in micronutrient absorption and production [46] and impacts overall health [47]. 

The menopausal transition is an important period for women’s bone health [48]. Since the CHNS did not record detailed information on women’s menopausal status, we did a stratified analysis by participants’ age. The case-control studies revealed that in women aged 60 years and below higher dietary diversity was inversely associated with the risk of fracture, but in women aged over 60 years a decreased risk of fracture was not observed. Similar phenomena were observed in other studies. In a cohort study conducted in Shanghai, China, high soy isoflavone intake reduced the risk of osteoporotic fractures in premenopausal and perimenopausal breast cancer survivors, but not in postmenopausal patients [49]. Women experienced accelerated bone mineral loss during menopausal transition [50], putting them at relatively higher risk of fracture [51]. Previous studies showed that calcium intake may reduce early postmenopausal bone loss [52] and fruits and vegetable related nutrients may protect against premenopausal bone loss [53]. However, after early postmenopausal stage, women gradually move onto the elderly stage, experiencing degeneration in digestive system and the consequent problems in absorption and usage of some nutrients [54]. Epidemiological studies showed digestive symptoms were more common in elderly women than in elderly men [55]. The changes in hormones and age-related degeneration in physical function might impact the body’s reaction to some nutrients [54,56]. Studies showed that serum calcium were lower in postmenopausal women than in pre-menopausal women and calcium supplements increase urinary calcium more than the calcium in serum among postmenopausal women [57,58,59,60]. We think these differences may partly explain the different associations of DDS with fracture between age groups in this study. Besides, the aging process is also characterized by degeneration of functional capacity, including balance, muscle strength, eyesight, etc. [61]. These factors may also increase the risk of fracture in the postmenopausal women. Considering the limited number of fracture cases in women aged over 60 years and missing information on supplement and medicine intake in our study, further studies are needed to explore this question more deeply.

Previous studies have already proved that some dietary patterns had favorable or unfavorable effects on bone health using a priori or data-driven methods [62]. However, the conclusions were inconsistent. A systematic review and meta-analysis of data-driven dietary patterns showed that adherence to the “healthy” pattern reduced the risk of fracture, whereas the “meat/Western” pattern increased the risk [63]. A cohort study of Singapore Chinese found that a meat-dim sum pattern [64], which was characterized by meat, dim sum, starchy food, and eggs, was not associated with hip fracture risk [65], and another prospective study in elderly Japanese indicated that a meat pattern might reduce the risk of fall-related fracture [66]. The inconsistencies between studies may be partly because dietary patterns with similar names in different studies could have great disparities in characteristic food groups. Compared with the assessment of dietary pattern, the DDS method is more suitable for comparisons across populations and more easily accepted by the public. The DDS method can be helpful in increasing attention to the risk of fracture and be relevant for prevention efforts at both individual and population levels.

In this study, we computed two DDSs, DDS-CDG and DDS-MDD-W. Compared with the DDS-CDG, the DDS-MDD-W gives special emphasis to dark green leafy vegetables and vitamin A-rich fruits and vegetables. Previous studies found that dark green, red, and yellow vegetables and dark-colored fruits had more pronounced beneficial effects on hip fracture than other fruits and vegetables, because they are the major sources of dietary phytochemicals, antioxidants, and vitamin K [67]. Besides, the DDS-MDD-W is a previously established score and thus more convenient for comparison across studies and populations. In this study, we found the DDS-CDG and DDS-MDD-W had a similar effect of decreased risk. This might be explained by the fact that the scores of dark green leafy vegetables were high in the studied population, and scores of vitamin A-rich fruit and vegetable were relatively low compared to the scores of total vegetables or dark green leafy vegetables. As a result, they did not generate great differentiation in total DDS-MDD-W compared with the DDS-CDG.

The strengths of this study include its prospective design, large sample size, and long follow-up period. In addition, cumulative means of DDS were used to assess long-term dietary exposure. However, it has several limitations. Firstly, the date of fracture was calculated from participants’ age when the fracture happened, which may cause recall bias. As this study only included first bond fracture and excluded participants who did not recall the precise age when the fracture happened, we believe the effect from recall bias was low in our analysis. Secondly, menopausal status and information on dietary supplement and medicine intake was not investigated in the CHNS. Women in early postmenopausal years experience accelerated bone mineral loss, which increases the risk of fracture, and usage of supplements or medicine such as hormones may change this trend. In this study, considering menopausal effects, we further grouped women by age of 60 years and subsequently performed a stratified analysis, which could partly make up for this defect.

## 5. Conclusions

Higher dietary diversity was associated with a decreased risk of fracture in men aged 40 years or older and in women aged 40 to 60 years in China. For older women, a decreased risk was not observed.

## Figures and Tables

**Table 1:** General characteristics of participants.

Characteristics	Men	Women
Number of participants	4795	5397
DDS-CDG	3.82 ± 0.98	3.81 ± 1.02
DDS-MDD-W	4.41 ± 1.07	4.41 ± 1.12
Age at entry (years)	51.4 ± 10.6	51.1 ± 10.7
Body mass index (kg/m^2^)	23.3 ± 3.4	23.5 ± 3.5
Region		
Southern China	59.0	59.5
Northern China	41.0	40.5
Residency		
Rural	60.0	60.2
Urban	40.0	39.8
Smoking status		
Smoker	59.5	4.1
Non-smoker	40.5	95.9
Physical activity level (MET-hours per week)		
<100	46.5	46.4
≥100	53.5	53.6
Education level		
Primary school and below	39.1	56.9
Middle school	31.4	23.2
High school and above	29.5	19.9
Income		
Low	30.3	30.7
Middle	31.1	32.1
High	38.6	37.2
Alcohol consumption ^1^		
Regular drinker	48.3	4.9
Non-regular drinker	51.7	95.1
Previous diagnosis of diabetes		
No	96.8	97.2
Yes	3.2	2.8

Values are means and standard deviations for continuous variables and percentages for categorical variables. DDS-CDG: dietary diversity score based on Chinese dietary guidelines; DDS-MDD-W: dietary diversity score based on Minimum Dietary Diversity for Women; MET: metabolic equivalent of task. ^1^ 149 missing values.

**Table 2:** Association between dietary diversity scores and incidence of fracture in men and women aged 40 years and above.

		DDS-CDG		DDS-MDD-W	
		HR (95% CI)	*p*	HR (95% CI)	*p*
Men	Crude	0.78 (0.65, 0.94)	0.008	0.75 (0.63, 0.89)	<0.001
	Adjusted	0.70 (0.56, 0.88)	0.002	0.67 (0.54, 0.82)	<0.001
Women	Crude	0.99 (0.86, 1.14)	0.905	0.97 (0.85, 1.11)	0.628
	Adjusted	0.94 (0.79, 1.12)	0.499	0.93 (0.79, 1.09)	0.371

Multivariate models were adjusted for age at entry (continuous), region (northern or southern China), residency (urban or rural), income (low, middle, or high), education level (primary school and below, middle school, or high school and above), body mass index (continuous), physical activity level (<100 or ≥100 metabolic equivalent of task-hours per week), smoking status (smoker or not), and previous diagnosis of diabetes (yes or no). DDS-CDG: dietary diversity score based on Chinese dietary guidelines; DDS-MDD-W: dietary diversity score based on Minimum Dietary Diversity for Women; HR: hazard ratio.

**Table 3:** Case control analyses of associations between dietary diversity scores and incidence of fracture in women aged 40 years and above.

	Number of Cases	DDS-CDG		DDS-MDD-W	
		OR (95% CI)	*p*	OR (95% CI)	*p*
≤60 years	143	0.74 (0.58, 0.95)	0.018	0.76 (0.60, 0.95)	0.016
>60 years	108	1.10 (0.83, 1.46)	0.495	1.00 (0.79, 1.27)	0.994

Cases and controls were matched (1:4) by age when fracture happened (a difference of no more than three years), region (southern or northern China), residency (urban or rural), and income (low, middle, or high). Conditional logistic regression models were used to estimate the association between the DDS and fracture, with adjustment of education level (primary school and below, middle school, or high school and above), smoking status (smoker or not), physical activity level (<100 or ≥100 metabolic equivalent of task-hours per week), and body mass index (continuous). DDS-CDG: dietary diversity score based on Chinese dietary guidelines; DDS-MDD-W: dietary diversity score based on Minimum Dietary Diversity for Women; OR: odds ratio.

## Supplementary Materials

The following are available online at https://www.mdpi.com/2072-6643/12/12/3655/s1, Table S1: Baseline characteristics of participants across dietary diversity score tertiles, Table S2: Dietary diversity scores across baseline characteristics in men and women, Table S3: Dietary diversity component scores between participants with and without bone fracture, Table S4: Sensitivity analysis of associations between dietary diversity scores and incidence of fracture.
