# Supplementary material for: Dietary Diversity and the Risk of Fracture in Adults: A Prospective Study
Source: Nutrients. 2020 Nov 27;12(12):3655. doi: 10.3390/nu12123655 (PMC7761242; doi:10.3390/nu12123655)
Supplement: Supplementary file 1 [file nutrients-12-03655-s001.zip › TableS1.docx]

**Table S1**. Baseline characteristics of participants across dietary diversity score tertiles.

|  | DDS-CDG | | |  | DDS-MDD-W | | |
| --- | --- | --- | --- | --- | --- | --- | --- |
|  | T1 | T2 | T3 |  | T1 | T2 | T3 |
| **Men** |  |  |  |  |  |  |  |
| Age at entry (years) | 51.2±10.5 | 50.7±10.4 | 52.4±10.8 |  | 51.5±10.6 | 50.4±10.3 | 52.5±10.7 |
| Body mass index (kg/m^2^) | 22.5±3.6 | 23.3±3.3 | 24.1±3.3 |  | 22.7±3.4 | 23.3±3.3 | 24.0±3.5 |
| Region |  |  |  |  |  |  |  |
| Southern China | 51.9 | 64.6 | 59.6 |  | 52.0 | 64.5 | 60.4 |
| Northern China | 48.1 | 35.4 | 40.4 |  | 48.0 | 35.5 | 39.6 |
| Residency |  |  |  |  |  |  |  |
| Rural | 84.7 | 62.5 | 34.7 |  | 81.5 | 61.9 | 36.3 |
| Urban | 15.3 | 37.5 | 65.3 |  | 18.5 | 38.1 | 63.7 |
| Smoking status |  |  |  |  |  |  |  |
| Smoker | 65.2 | 61.5 | 51.9 |  | 64.8 | 62.1 | 51.3 |
| Non-Smoker | 34.8 | 38.5 | 48.1 |  | 35.2 | 37.9 | 48.7 |
| Physical activity level (MET-hours per week) |  |  |  |  |  |  |  |
| <100 | 35.8 | 46.9 | 55.9 |  | 37.8 | 46.9 | 54.9 |
| ≥100 | 64.2 | 53.1 | 44.1 |  | 62.2 | 53.1 | 45.1 |
| Education level |  |  |  |  |  |  |  |
| Primary school and below | 59.0 | 39.9 | 20.0 |  | 56.3 | 40.6 | 20.2 |
| Middle school | 28.2 | 34.6 | 30.9 |  | 29.3 | 33.0 | 31.9 |
| High school and above | 12.8 | 25.5 | 49.1 |  | 14.4 | 26.4 | 47.9 |
| Income |  |  |  |  |  |  |  |
| Low | 53.5 | 28.1 | 11.0 |  | 48.6 | 28.0 | 14.0 |
| Middle | 30.7 | 36.4 | 25.9 |  | 32.6 | 34.6 | 26.0 |
| High | 15.8 | 35.5 | 63.1 |  | 18.8 | 37.4 | 60.0 |
| Alcohol consumption ^1^ |  |  |  |  |  |  |  |
| Regular drinker | 45.9 | 50.1 | 48.6 |  | 46.3 | 51.4 | 47.2 |
| Non-regular drinker | 54.1 | 49.9 | 51.4 |  | 53.7 | 48.6 | 52.8 |
| Previous diagnosis of diabetes |  |  |  |  |  |  |  |
| No | 98.4 | 97.2 | 94.9 |  | 98.0 | 97.2 | 95.2 |
| Yes | 1.6 | 2.8 | 5.1 |  | 2.0 | 2.8 | 4.8 |
| **Women** |  |  |  |  |  |  |  |
| Age at entry (years) | 51.3±10.9 | 50.5±10.8 | 51.6±10.2 |  | 51.6±11.2 | 50.3±10.5 | 51.5±10.3 |
| Body mass index (kg/m^2^) | 23.0±3.5 | 23.5±3.5 | 24.1±3.6 |  | 23.0±3.5 | 23.5±3.5 | 24.0±3.5 |
| Region |  |  |  |  |  |  |  |
| Southern China | 54.5 | 65.3 | 58.8 |  | 52.8 | 65.3 | 60.4 |
| Northern China | 45.5 | 34.7 | 41.2 |  | 47.2 | 34.7 | 39.6 |
| Residency |  |  |  |  |  |  |  |
| Rural | 83.1 | 61.5 | 36.1 |  | 82.0 | 61.0 | 36.7 |
| Urban | 16.9 | 38.5 | 63.9 |  | 18.0 | 39.0 | 63.3 |
| Smoking status |  |  |  |  |  |  |  |
| Smoker | 5.2 | 4.1 | 3.1 |  | 5.8 | 3.6 | 2.9 |
| Non-Smoker | 94.8 | 95.9 | 96.9 |  | 94.2 | 96.4 | 97.1 |
| Physical activity level (MET-hours per week) |  |  |  |  |  |  |  |
| <100 | 38.0 | 49.4 | 51.8 |  | 40.0 | 48.7 | 50.5 |
| ≥100 | 62.0 | 50.6 | 48.2 |  | 60.0 | 51.3 | 49.5 |
| Education level |  |  |  |  |  |  |  |
| Primary school and below | 78.4 | 59.9 | 32.6 |  | 76.4 | 59.2 | 34.2 |
| Middle school | 15.9 | 24.6 | 29.1 |  | 16.9 | 24.5 | 28.5 |
| High school and above | 5.7 | 15.5 | 38.3 |  | 6.7 | 16.3 | 37.3 |
| Income |  |  |  |  |  |  |  |
| Low | 51.5 | 29.0 | 11.6 |  | 48.5 | 29.0 | 14.0 |
| Middle | 32.1 | 37.6 | 26.8 |  | 32.9 | 36.7 | 26.6 |
| High | 16.4 | 33.4 | 61.6 |  | 18.6 | 34.3 | 59.4 |
| Alcohol consumption ^2^ |  |  |  |  |  |  |  |
| Regular drinker | 5.0 | 4.3 | 5.4 |  | 4.9 | 4.7 | 5.1 |
| Non-regular drinker | 95.0 | 95.7 | 94.6 |  | 95.1 | 95.3 | 94.9 |
| Previous diagnosis of diabetes |  |  |  |  |  |  |  |
| No | 98.8 | 97.3 | 95.7 |  | 98.7 | 97.3 | 95.6 |
| Yes | 1.2 | 2.7 | 4.3 |  | 1.3 | 2.7 | 4.4 |

Values are means and standard deviations for continuous variables and percentages for categorical variables. DDS-CDG: dietary diversity score based on Chinese dietary guidelines; DDS-MDD-W: dietary diversity score based on Minimum Dietary Diversity for Women; MET: metabolic equivalent of task. Dietary diversity scores were grouped into tertiles from low to high (T1, T2, T3). ^1^ 70 participants were dropped for missing information on alcohol consumption. ^2^ 79 participants were dropped for missing information on alcohol consumption.
