# Supplementary material for: Dietary Diversity and the Risk of Fracture in Adults: A Prospective Study
Source: Nutrients. 2020 Nov 27;12(12):3655. doi: 10.3390/nu12123655 (PMC7761242; doi:10.3390/nu12123655)
Supplement: Supplementary file 1 [file nutrients-12-03655-s001.zip › TableS2.docx]

**Table S2.** Dietary diversity scores across baseline characteristics in men and women.

| Characteristics | Men (n=4,795) | |  | Women (n=5,397) | |
| --- | --- | --- | --- | --- | --- |
|  | DDS-CDG | DDS-MDD-W |  | DDS-CDG | DDS-MDD-W |
| Age at entry (years) |  |  |  |  |  |
| 40-50 | 3.76±0.88 | 4.34±0.95 |  | 3.76±0.93 | 4.36±1.00 |
| 50-60 | 3.88±1.04 | 4.46±1.10 |  | 3.92±1.11 | 4.53±1.23 |
| Over 60 | 3.91±1.13 | 4.52±1.28 |  | 3.82±1.11 | 4.41±1.26 |
| Region |  |  |  |  |  |
| Southern China | 3.87±0.96 | 4.46±1.02 |  | 3.84±1.00 | 4.45±1.07 |
| Northern China | 3.75±1.01 | 4.33±1.14 |  | 3.75±1.03 | 4.34±1.17 |
| Residency |  |  |  |  |  |
| Rural | 3.47±0.81 | 4.04±0.87 |  | 3.45±0.83 | 4.04±0.90 |
| Urban | 4.34±0.97 | 4.96±1.10 |  | 4.34±1.04 | 4.97±1.17 |
| Education level |  |  |  |  |  |
| Primary school and below | 3.39±0.80 | 3.97±0.86 |  | 3.44±0.82 | 4.02±0.89 |
| Middle school | 3.83±0.88 | 4.43±0.97 |  | 4.05±0.97 | 4.65±1.09 |
| High school and above | 4.37±1.01 | 4.98±1.14 |  | 4.59±1.04 | 5.22±1.20 |
| Income |  |  |  |  |  |
| Low | 3.26±0.77 | 3.89±0.87 |  | 3.25±0.77 | 3.89±0.87 |
| Middle | 3.71±0.81 | 4.29±0.91 |  | 3.72±0.88 | 4.30±0.98 |
| High | 4.34±0.98 | 4.91±1.11 |  | 4.34±1.03 | 4.93±1.17 |
| Smoking status |  |  |  |  |  |
| Smoker | 3.72±0.93 | 4.29±0.99 |  | 3.57±0.92 | 4.07±1.04 |
| Non-Smoker | 3.97±1.03 | 4.58±1.16 |  | 3.82±1.02 | 4.42±1.12 |
| Alcohol consumption ^1^ |  |  |  |  |  |
| Regular drinker | 3.83±0.94 | 4.40±1.01 |  | 3.82±1.03 | 4.45±1.16 |
| Non-regular drinker | 3.81±1.02 | 4.41±1.12 |  | 3.81±1.02 | 4.41±1.11 |
| Physical activity level (MET-hours per week) |  |  |  |  |  |
| <100 | 3.99±0.98 | 4.57±1.09 |  | 3.90±0.98 | 4.47±1.10 |
| ≥100 | 3.67±0.95 | 4.27±1.03 |  | 3.73±1.04 | 4.35±1.12 |
| Body mass index (kg/m^2^) |  |  |  |  |  |
| Below 24 | 3.67±0.95 | 4.27±1.03 |  | 3.71±0.99 | 4.31±1.09 |
| 24-28 | 4.05±0.98 | 4.63±1.09 |  | 3.94±1.03 | 4.55±1.13 |
| 28 or higher | 4.09±0.95 | 4.65±1.05 |  | 3.97±1.02 | 4.56±1.15 |
| History of diabetes |  |  |  |  |  |
| No | 3.80±0.97 | 4.39±1.06 |  | 3.79±1.01 | 4.39±1.10 |
| Yes | 4.34±1.05 | 4.95±1.20 |  | 4.35±1.10 | 5.09±1.38 |

Values were presented as means and standard deviations. Difference across groups were compared by *t*-test or one-way ANOVA. All variables were associated with both DDSs with *p*<0.001 for difference across categories, except for DDS-CDG across regions in women (*p*=0.001), DDS-CDG across alcohol consumption categories (*p*=0.461 in men; *p*=0.845 in women), DDS-MDD-W across alcohol consumption categories (*p*=0.840 in men; *p*=515 in women). DDS-CDG: dietary diversity score based on Chinese dietary guidelines; DDS-MDD-W: dietary diversity score based on Minimum Dietary Diversity for Women; MET: metabolic equivalent of task. ^1^149 participants were dropped for missing information on alcohol consumption.
