# Supplementary material for: Dietary Diversity and the Risk of Fracture in Adults: A Prospective Study
Source: Nutrients. 2020 Nov 27;12(12):3655. doi: 10.3390/nu12123655 (PMC7761242; doi:10.3390/nu12123655)
Supplement: Supplementary file 1 [file nutrients-12-03655-s001.zip › TableS3.docx]

**Table S3.** Dietary diversity component scores between participants with and without bone fracture.

|  | Men | | |  | Women | | |
| --- | --- | --- | --- | --- | --- | --- | --- |
|  | No | Yes | *p* |  | No | Yes | *p* |
| Number of participants | 4624 | 171 |  |  | 5146 | 251 |  |
| **DDS-CDG** |  |  |  |  |  |  |  |
| Cereals and tubers | 1.00(1.00,1.00) | 1.00(1.00,1.00) | 0.892 |  | 1.00(1.00,1.00) | 1.00(1.00,1.00) | 0.259 |
| Vegetables | 1.00(1.00,1.00) | 1.00(1.00,1.00) | 0.059 |  | 1.00(1.00,1.00) | 1.00(1.00,1.00) | 0.596 |
| Fruits | 46.41 | 25.15 | <0.001 |  | 52.97 | 37.45 | <0.001 |
| Meat | 0.67(0.33,1.00) | 0.67(0.29,0.89) | 0.009 |  | 0.67(0.33,0.93) | 0.67(0.22,0.89) | 0.037 |
| Soybeans and nuts | 0.33(0.17,0.56) | 0.33(0.08,0.57) | 0.933 |  | 0.33(0.13,0.53) | 0.33(0.12,0.53) | 0.659 |
| Eggs | 0.33(0.11,0.58) | 0.25(0.00,0.42) | <0.001 |  | 0.33(0.11,0.56) | 0.33(0.00,0.50) | 0.031 |
| Aquatic products | 60.16 | 47.37 | 0.001 |  | 58.41 | 54.18 | 0.184 |
| Milk and dairy products | 18.64 | 7.60 | <0.001 |  | 20.29 | 15.14 | 0.047 |
| **DDS-MDD-W** |  |  |  |  |  |  |  |
| Starchy staples | 1.00(1.00,1.00) | 1.00(1.00,1.00) | 0.734 |  | 1.00(1.00,1.00) | 1.00(1.00,1.00) | 0.184 |
| Pulses | 0.33(0.16,0.53) | 0.33(0.00,0.56) | 0.790 |  | 0.33(0.13,0.50) | 0.33(0.12,0.56) | 0.552 |
| Nuts and seeds | 24.74 | 18.13 | 0.048 |  | 25.09 | 20.32 | 0.088 |
| Dairy | 18.64 | 7.60 | <0.001 |  | 20.29 | 15.14 | 0.047 |
| Eggs | 0.33(0.11,0.58) | 0.25(0.00,0.42) | <0.001 |  | 0.33(0.11,0.56) | 0.33(0.00,0.50) | 0.031 |
| Dark green leafy vegetables | 0.87(0.67,1.00) | 0.83(0.67,1.00) | 0.559 |  | 0.83(0.67,1.00) | 0.92(0.67,1.00) | 0.008 |
| Other vitamin A-rich fruits and vegetables | 0.20(0.00,0.33) | 0.11(0.00,0.33) | 0.008 |  | 0.20(0.00,0.33) | 0.11(0.00,0.33) | <0.001 |
| Other vegetables | 0.67(0.44,0.83) | 0.60(0.33,0.78) | 0.006 |  | 0.67(0.44,0.83) | 0.67(0.33,0.83) | 0.417 |
| Other fruits | 43.43 | 22.81 | <0.001 |  | 49.65 | 33.07 | <0.001 |
| Meat, poultry, and fish | 0.83(0.47,1.00) | 0.67(0.33,1.00) | 0.019 |  | 0.75(0.40,1.00) | 0.75(0.33,1.00) | 0.105 |

For food groups with less than 50% of consumers, component scores were recorded as binary, and the percentages of consumers were present; otherwise, medians and quartiles of component scores were shown. Differences between groups were tested by chi-square tests for categorical variables and Wilcoxon tests for continuous variables. DDS-CDG: dietary diversity score based on Chinese dietary guidelines; DDS-MDD-W: dietary diversity score based on Minimum Dietary Diversity for Women.
