# Supplementary material for: Dietary Diversity and the Risk of Fracture in Adults: A Prospective Study
Source: Nutrients. 2020 Nov 27;12(12):3655. doi: 10.3390/nu12123655 (PMC7761242; doi:10.3390/nu12123655)
Supplement: Supplementary file 1 [file nutrients-12-03655-s001.zip › TableS4.docx]

**Table S4.** Sensitivity analysis of associations between dietary diversity scores and incidence of fracture.

|  |  | DDS-CDG | |  | DDS-MDD-W | |
| --- | --- | --- | --- | --- | --- | --- |
|  |  | HR (95%CI) | *p* |  | HR (95%CI) | *p* |
| Men | Model 1 | 0.72(0.57,0.90) | 0.005 |  | 0.69(0.56,0.85) | <0.001 |
|  | Model 2 | 0.73(0.58,0.93) | 0.009 |  | 0.69(0.56,0.86) | 0.001 |
| Women | Model 1 | 0.92(0.77,1.10) | 0.370 |  | 0.91(0.77,1.07) | 0.261 |
|  | Model 2 | 1.01(0.84,1.22) | 0.891 |  | 1.00(0.85,1.19) | 0.968 |

Multivariate models were adjusted for age at entry (continuous), region (northern or southern China), residency (urban or rural), income (low, middle, or high), education level (primary school and below, middle school, or high school and above), body mass index (continuous), physical activity level (<100 or ≥100 metabolic equivalent of task-hours per week), smoking status (smoker or not), previous diagnosis of diabetes (yes or no), alcohol consumption (regular drinker or not; only in model 1), and wave at entry (1997, 2000, 2004, 2006, 2009, or 2011; only in model 2). In model 1, 149 participants were dropped for missing information on alcohol consumption. DDS-CDG: dietary diversity score based on Chinese dietary guidelines; DDS-MDD-W: dietary diversity score based on Minimum Dietary Diversity for Women; HR: hazard ratio.
